# Milrinone in Enterovirus 71 Brain Stem Encephalitis

**DOI:** 10.3389/fphar.2016.00082

**Published:** 2016-03-29

**Authors:** Shih-Min Wang

**Affiliations:** ^1^Department of Pediatrics, National Cheng Kung University Hospital, College of Medicine, National Cheng Kung UniversityTainan, Taiwan; ^2^Department of Emergency Medicine, National Cheng Kung University Hospital, College of Medicine, National Cheng Kung UniversityTainan, Taiwan; ^3^Center of Infectious Disease and Signaling Research, National Cheng Kung UniversityTainan, Taiwan

**Keywords:** Enterovirus 71, brain stem encephalitis, pulmonary edema, milrinone, inflammation

## Abstract

Enterovirus 71 (EV71) was implicated in a widespread outbreak of hand-foot-and-mouth disease (HFMD) across the Asia Pacific area since 1997 and has also been reported sporadically in patients with brain stem encephalitis. Neurogenic shock with pulmonary edema (PE) is a fatal complication of EV71 infection. Among inotropic agents, milrinone is selected as a therapeutic agent for EV71- induced PE due to its immunopathogenesis. Milrinone is a type III phosphodiesterase inhibitor that has both inotropic and vasodilator effects. Its clinical efficacy has been shown by modulating inflammation, reducing sympathetic over-activity, and improving survival in patients with EV71-associated PE. Milrinone exhibits immunoregulatory and anti-inflammatory effects in the management of systemic inflammatory responses in severe EV71 infection.

## Epidemiology and Clinical Spectrum

Enteroviruses can result in a wide range of clinical illnesses, including herpangina, hand-foot-and-mouth disease (HFMD), and occasionally causes neurological complications, meningitis, encephalitis and poliomyelitis-like paralysis. Enterovirus 71 (EV71), which was first identified in 1969, causes meningitis (Schmidt et al., 1974). In Taiwan the initial outbreak of EV71 infection of 1998, total number of cases estimated at around 1.5 million, among which 405 had severe diseases and 78 died (Ho et al., 1999). Approximately 30% of hospitalized HFMD patients with EV71 infection developed more complicated disease (Chang et al., 1999) including aseptic meningitis, acute flaccid paralysis, brain stem encephalitis (BE), autonomic nervous system (ANS) dysregulation, and pulmonary edema (PE), which in some cases can be fatal (Wang et al., 1999, 2003; Wang and Liu, 2009, 2014).

## Management

### Epinephrine

For the classical vasopressor–inotropes, such as epinephrine, the mechanisms of increasing myocardial contractility are primarily based on stimulating α- or β-adrenergic receptors located on the cell membrane of myocardial cells. Epinephrine exerts its cardiovascular effects by stimulating the α- or β-adrenergic receptors in a dose-dependent manner. At high doses, epinephrine also stimulates the vascular and cardiac α1-receptors causing vasoconstriction and increased inotropy. The net hemodynamic effects of epinephrine administration are significant increases in blood pressure and systemic blood flow. EV71-infected patients in the phase of ANS dysregulation display hypertension, tachycardia, and severe peripheral vasoconstriction. Once the surge of sympathetic over-activity resulted in PE, the cardiopulmonary failure followed. The plasma levels of epinephrine in EV71-infected patients with ANS dysregulation and PE were markedly high (Liao et al., 2015). Furthermore, the percentages EV71-infected cells were increased in THP-1 and Jurkat cells via treatment with epinephrine. At least twofold increase in virus titer was observed in EV71-infected A549, SK-N-SH, and hPBMCs after treatment with epinephrine (Liao et al., 2015). Furthermore, catecholamines have many disadvantages, including increased myocardial oxygen consumption, heart rate, afterload, and the risk of dysrhythmias (Wessel, 2001). Therefore, epinephrine is contraindicated in these patients.

### Milrinone

Patients with EV71 associated PE have a high case-fatality rate around 80–90%. Without prompt management, the fatality may occur within 6–12 h after onset of BE. Milrinone, a bipyridine derivative and phosphodiesterase (PDE) III inhibitor, has both inotropic and vasodilatation characters. Milrinone enhances myocardial contractility, promotes myocardial relaxation, and decreases vascular tone in the systemic and pulmonary vascular beds without excessive increases in myocardial oxygen consumption (Shipley et al., 1996). It exerts its cardiovascular effects through the inhibition of cyclic adenosine 3′,5′-monophosphate (cAMP) degradation but have no interaction with the adrenergic receptors nor do they inhibit Na-K ATPase. Inhibition of cAMP degradation by intracellular PDE3 may attenuate inflammation, reduce edema formation, improve endothelial function, and induce pulmonary vasodilation (Hayashida et al., 1999). A pilot study was designed to evaluate the potential therapeutic effects of milrinone in the treatment of patients with EV71-induced PE (Wang et al., 2005). The mortality rate, sympathetic activity and inflammatory cells were significantly lower in patients with milrinone treatment than patients without milrinone treatment.

### Clinical Outcome

Meyer et al. (2011) reviewed the randomized and quasi-controlled trials of milrinone treatment in children with low cardiac output syndrome after congenital heart surgery (Hoffman et al., 2003; Cai et al., 2008) and in non-hyperdynamic septic shock (Barton et al., 1996; Lindsay et al., 1998). They concluded these trials have shown significantly improved cardiovascular function, however, none of the studies demonstrated improved long-term survival and reduced long-term morbidity (Meyer et al., 2011). In a randomized, controlled study, the 1-week mortality of patients with EV71-associated PE was significantly lower in patients with milrinone treatment than patients without milrinone treatment. The median duration of ventilator-free days was also longer in the milrinone treatment group (Chi et al., 2013). The effectiveness and efficacy of milrinone treatment in patients with EV71 associated PE has been proven in historically controlled (Wang et al., 2005) and randomized controlled studies (Chi et al., 2013).

### Immunoregulation

Phosphodiesterase inhibitors can regulate the activity of immune cells by increasing intracellular levels of cyclic nucleotides. Milrinone was administered to mice either once or five times at 24 h intervals and resulted in decreased percentages of B cells (CD19^+^) and increased percentages of T cells (CD3^+^, CD4^+^, CD8^+^) in splenocyte subpopulations (Szczypka and Obmiñska-Mrukowicz, 2010). The expression frequency of regulatory T cell (Tregs) profiles CD4^-^CD8^-^, CD4^+^Foxp3^+^, and CD4^+^CD25^+^Foxp3^+^ decreased significantly in the advanced stages of EV71 infection, in patients with ANS dysregulation or PE. Fu et al. (2009) suggested the decreased CD4^+^CD25^+^Foxp3^+^expression frequency in severe EV71 infection may associate with the decreased plasma level of TGF-β and increased plasma level of IL-6. Plasma concentrations of cAMP were significantly decreased in patients with EV71-induced ANS dysregulation or PE compared with patients with EV71-induced HFMD or BE. The modulating effect of PDE inhibitors on lymphocyte subpopulations is related to their influence on synthesis and release of cytokines (Szczypka and Obmiñska-Mrukowicz, 2010). Intravenous administration of milrinone reduced the levels of serum pro-inflammatory cytokines in patients in a prospective randomized clinical study (Hayashida et al., 1999). cAMP has been considered to have general anti-inflammatory activity, can inhibit inflammation-related chemotaxis, lysosomal enzyme and histamine release, mitogenesis, and lymphocyte-mediated cytotoxicity (Moore and Willoughby, 1995). Milrinone has direct effect on decreasing the production of pro-inflammatory cytokines by elevating cAMP and inhibiting the NF-κB pathway (Barin and Čiháková, 2013). Milrinone therapy not only increased the expression frequency of CD4^+^Foxp3^+^ and plasma levels of cAMP in severe EV71 infection but also reduced the plasma levels of cytokines, IL-6, IL-8, IL-10, and IL-13 (Wang et al., 2005, 2014).

### Challenges

Milrinone provides a useful therapeutic approach for treating life threatening EV71 infections. A low-dose β-blocker in combination with milrinone improved cardiac function in acute decompensated heart failure patients with tachycardia (Kobayashi et al., 2012). Liao et al. (2015) also showed β-blocker reduced the percentages of EV71-infected cells in THP-1, Jurkat cells and hPBMCs with norepinephrine and epinephrine treatment. The combination of β-blockade with PDE III inhibition is counterintuitive; they have opposing effects on intracellular cAMP concentrations. A pharmacological clamp is effectively applied to the heart, holding it at a given contractility and rate, opposing fluctuations in endogenous catecholamines (McNeilly, 2014). Acute β-blockade in the presence of a PDE III inhibitor has been shown to be feasible and suggested as cardioprotective in sepsis (Schmittinger et al., 2008; Morelli et al., 2013). Therefore, standard therapy with milrinone

in patients EV71-induced PE, β-blocker may be considered as an adjunct therapy.

## Author Contributions

S-MW: Conceived the study and wrote the paper.

## Conflict of Interest Statement

The author declares that the research was conducted in the absence of any commercial or financial relationships that could be construed as a potential conflict of interest.
